# Unknown variant of the accessory subscapularis muscle?

**DOI:** 10.1007/s12565-021-00633-8

**Published:** 2021-09-30

**Authors:** Nicol Zielinska, R. Shane Tubbs, Marko Konschake, Łukasz Olewnik

**Affiliations:** 1 Department of Anatomical Dissection and Donation, Medical University of Lodz, Lodz, Poland; 2 Department of Neurosurgery, Tulane University School of Medicine, New Orleans, LA USA; 3 Department of Neurosurgery and Ochsner Neuroscience Institute, Ochsner Health System, New Orleans, LA USA; 4 Department of Anatomical Sciences, St. George’s University, West Indies, Grenada; 5 Department of Neurology, Tulane University School of Medicine, New Orleans, LA USA; 6 Department of Structural and Cellular Biology, Tulane University School of Medicine, New Orleans, LA USA; 7 Department of Surgery, Tulane University School of Medicine, New Orleans, LA USA; 8 Institute of Clinical and Functional Anatomy, Medical University of Innsbruck (MUI), Innsbruck, Austria

**Keywords:** Accessory subscapularis muscle, Subscapularis muscle, Case report, Compression, Autograft, Stabilization

## Abstract

Acting in medial rotation of the arm, the subscapularis (SM) is the most powerful and largest muscle of the rotator cuff. It is morphologically variable, especially in the number of tendons, place of insertion, and number of bellies, and it is sometimes fused with another muscle. An accessory subscapularis muscle (ASM) is among the morphological variations of the SM, but it is a really rare variant. The present case describes a very rare ASM that is divided into proximal tendinous attachment, intermediate fleshy muscular belly and distal tendinous attachment. Its origin is located on the lateral border of the scapula, but some fibers are connected with the muscular part of the SM. Its distal attachment is fused with the capsule of shoulder joint, above the tendinous insertion of the SM. Such an arrangement allows for greater stabilization of the joint. Moreover, there is a possibility that it could be used during treatment of ruptured SM tendons.

## Introduction

The most powerful and largest muscle of the rotator cuff is the subscapularis (SM), which acts in medial rotation of the arm (Bergman et al. 2017). The SM consists of two parts: muscular and tendinous. Its origin, which is represented by the muscle belly, is on the costal surface of the scapula, called the subscapularis fossa. Its distal attachment (a terminal portion of the tendinous slip) is on the superior part of the humerus. The insertion of the SM is morphologically variable (Zielinska et al. 2021c). The blood supply to the SM is usually provided by the subscapular artery, which is a branch of the axillary artery (Maruvada et al. 2020). The SM is innervated by the upper and lower subscapular nerves, which arise from the posterior cord of the brachial plexus (Kasper et al. 2008).

As mentioned above, the main function of the SM is medial rotation of the arm. In certain positions, it also functions in adduction and extension. In view of its structure and distal attachment, it also participates in stabilizing the glenohumeral joint (Nguyen and Duong 2020; Zielinska et al. 2021a, c).

The SM is morphologically variable. For example, the insertion can vary in number of tendons, or in the placement of attachment (Zielinska et al. 2021b). Another variation is the occurrence of an accessory subscapularis muscle (ASM) (Breisch 1986; Gruber 1859; Kameda 1976; MacAlister 1875; Staniek and Brenner 2012; Zielinska et al. 2021a). The origin of the SM is always located on the anterior surface of the scapula, but there are variations in which this part of the muscle is fused with another muscle such as the latissimus dorsi or teres major (Kellam et al. 2019).

An additional structure is usually related to compression on certain neural or vascular structures. For example, an ASM can be associated with quadrilateral space syndrome (Krause and Youdas 2017). Patients usually suffer from pain and loss of sensation in the shoulder region (Hangge et al. 2018).

The present case describes a very rare ASM. Structurally, it composed of proximal tendinous attachment, intermediate fleshy muscular belly and distal tendinous attachment. The tendinous origin was located on the lateral border of the scapula, but some proximal fibers were connected to the muscular part of the SM. Next, it passed into the muscular part, and then into distal attachment fused with the capsule of the shoulder joint above the insertion of the SM. We think that this structure, if present, could be helpful during operative treatment of a SM tendon rupture, so knowledge about it should be important for orthopedics specialists or surgeons.

## Case report

A 73-year-old at death female cadaver was subjected to routine anatomical dissection for research and teaching purposes at the Department of Anatomical Dissection and Donation, Medical University of Lodz, Poland. The right upper limb underwent traditional anatomical dissection and a morphological variant of the ASM was found (Olewnik et al. 2021, 2020; Podgórski et al. 2019; Szewczyk et al. 2021; Zielinska et al. 2021a, c).

The next stage of the investigation involved a detailed assessment of this structure. The ASM consisted of proximal tendinous attachment, intermediate fleshy muscular belly and distal tendinous attachment. Its proximal tendinous part originated from the lateral border of the scapula, but some of the proximal fibers were connected with the muscular part of the SM. At that point, the width was 6.45 mm and the thickness was 0.29 mm. The length of this tendinous part (from the origin to the myotendinous junction) was 31.09 mm. The first (proximal) myotendinous junction was 3.51 mm wide and 0.72 mm thick.

Next, the ASM passed into the muscular part, with length 73.56 mm. This part ended at the second (distal) myotendinous junction and the distal attachment was represented by a tendinous slip. At that point, the width was 3.74 mm and the thickness was 0.74 mm. The distal attachment was 45.83 mm long. It is worth mentioning that the tendinous portion expanded. The point of greatest expansion was 14.75 mm from the insertion; the width here was 2.10 mm and the thickness was 0.87 mm.

**Fig. 1:**
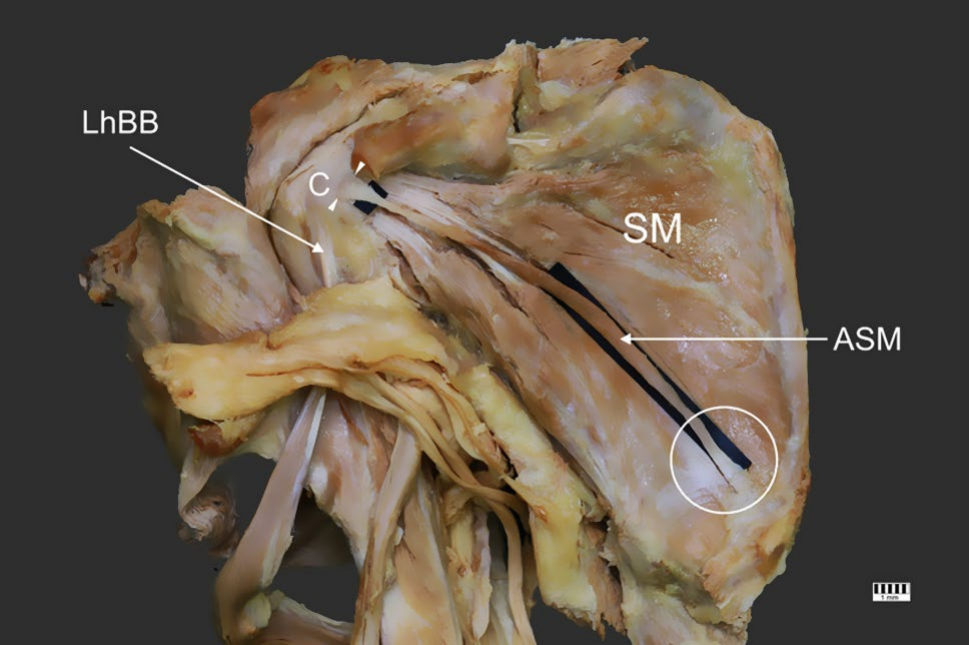
Accessory subscapularis muscle. *LhBB* long head of the biceps brachii; *SM* subscapularis muscle; *C* capsule; *ASM* accessory subscapularis muscle; white arrowheads indicate the proximal attachment; white circle indicates the distal attachment

As mentioned above, the distal attachment was fused with the medial part of the capsule of the shoulder joint, above the insertion of the SM. It was 7.84 mm wide and 1.64 mm thick (Fig. 1).

An electronic caliper (Mitutoyo Corporation, Kawasaki-shi, Kanagawa, Japan) was used for these measurements. Each measurement was repeated twice with an accuracy of up to 0.1 mm.

The muscle was innervated by small branch from the lower subscapular nerve, a component of the posterior cord of the brachial plexus that also innervated the SM. The vascularization was also the same for both the ASM and SM. The latter was supplied by a small arteriole arising from the subscapular artery, which is a large branch of the axillary artery.

During dissection of the upper limb, the fascia of the SM was removed to reveal the SM. No other morphological variabilities were found. The morphometric measurements of individual parts of the ASM are given in Table 1.

**Table 1:** Morphometric measurements of individual parts of the accessory subscapularis muscle

	Proximal tendinous attachment	Intermediate muscular belly	Distal tendinous attachment
Length	31.09 mm	73.56 mm	45.83 mm
Width			
Proximally	6.43 mm (PA)	3.51 mm(PMJ)	3.74 mm (DMJ)
Distally	3.51 mm (PMJ)	3.74 mm (DMJ)	2.10 mm (ExP)
ExP	–	–	7.84 mm (DA)
Thickness			
Proximally	0.29 mm (PA)	0.72 mm (PMJ)	0.74 mm (DMJ)
Distally	0.72 mm (PMJ)	0.74 mm (DMJ)	1.64 mm (DA)
ExP	–	–	0.87 mm (ExP)

*PA* proximal attachment, *PMJ* proximal myotendinous junction, *DMJ* distal myotendinous junction, *DA* distal attachment, *ExP* point of greatest expansion

## Discussion

The SM is morphologically variable in both its insertion and its origin. Sometimes, its proximal attachment is fused with another muscle such as the latissimus dorsi or teres minor (Kellam et al. 2019). As mentioned above, the numbers of tendons or bellies, or the place of insertion, can vary. For example, Zielinska et al. (2021c) created a classification system based on the number of tendons, which ranged from one to eight. Four types were distinguished, the fourth being divided into five subtypes. Interestingly, the authors also showed that the insertion is always located on the superior part of the humerus, but the tendon or tendons could be attached in different places, for example the lesser tuberosity (its entire surface, or inferomedial part, or medial part, or its crest), greater tuberosity, intertubercular groove, or medial border of the surgical neck just below the lesser tubercle of the humerus (Zielinska et al. 2021c).

Another morphological variation is the ASM. Several cases of the ASM have been described in the literature. Gruber (1859) was the first author to report the structure; he called it the subscapularis minor muscle or marginal axillary bundle, depending on the degree of fusion with the SM. The origin was the same as in the present case: the lateral border of the scapula. However, the point of insertion differed. The ASM described by Gruber (1859) was attached to the lesser tuberosity; in our case, it was fused with the capsule of the shoulder joint.

There was a similar situation in cases described by MacAlister (1875), who distinguished two types of ASM: a subscapulocapsularis muscle and a subscapulohumeral muscle. Each had an origin on the lateral border of the scapula, and this is a common feature; but the point of insertion differed from our case. The subscapulohumeral muscle inserted to the lesser tuberosity and the crest of the lesser tuberosity. The subscapulocapsularis inserted to the neck of the humerus and the lower part of the transverse humeral ligament (MacAlister 1875).

Staniek and Berner (2012) also described a muscle originating from the lateral border of the scapula, called the infraglenoid muscle; but it was in the upper third, so it would be a mistake to compare it with the present case.

Yoshinaga et al. (2008) described an ASM in which the tendinous insertion was fused with the capsule of the shoulder joint. The distal attachment was the same as in the present case, but the location of the proximal attachment differed; again, therefore, we cannot say that the courses of these structures are the same.

Looking in the literature for a type of ASM identical to ours, we found cases described by Breisch (1986), Kameda (1976), and Zielinska et al. (2021a). However, they all differed from our findings, so we can conclude that this is the first description of such a case.

The occurrence of an additional structure is usually related to compression, for example of neural or vascular elements.

The ASM can also compress some structures. Zielinska et al. (2021a) found a case in which the ASM was located above the SM, and between the two muscles was a posterior cord from which nerves arose. Breisch (1986), Kameda (1976), and Yoshinaga et al. (2008) described a similar situation, which predisposes to some kind of neuropathy. For instance, it can lead to quadrilateral space syndrome, the main symptoms of which are weakness and disability of teres minor and deltoid muscles. Since these muscles are responsible for abduction and external rotation, those movements are likely to be limited. Moreover, patients usually suffer from pain localized in the shoulder region and loss of sensation in the lateral region of the shoulder (Hangge et al. 2018; Krause and Youdas 2017).

However, not all types of ASM cause compression. In the present case, the ASM was separated from the SM by the fascia of the latter. As mentioned above, the ASM originated from the lateral border of the scapula as a single independent tendon, but some tendinous fibers in the small proximal part were connected to the muscular fibers of the SM. The tendinous insertion of the ASM was also independent and the distal attachment point differed from that of the SM. Interestingly, there were no neural or vascular structures between the SM and the ASM; the posterior cord and the nerves arising from it were placed above both muscles. The same applied to the posterior humeral circumflex artery. Therefore, we can conclude that no pathologies resulting from compression, such as quadrilateral space syndrome, could be connected to this additional structure.

Since there seemed to be no potential disadvantages, we started looking for advantages of this ASM. We hypothesize that it can be used as a source of material during an autograft, as in the case of an additional extensor hallucis longus (EHL). Kurishige (2019) used the extensor hallucis capsularis (a type of additional EHL) in treating EHL rupture. The effectiveness was assessed 1 year after the operation. Significant improvements in strength of the EHL, its activity, and foot stabilization were noted (Kurashige 2019). Of course, other muscles could have been used in such a case. The EDL tendon, the gracilis, fibularis longus muscle, and the semitendinosus could also help. However, the procedure would be invasive and the original function of the muscle could be sacrificed (Olewnik 2019; Olewnik et al. 2019; So et al. 2018).

The literature contains no account of the use of an ASM as autograft in treating rupture of the SM tendons, but in view of the opportunities that an autograft with an additional EHL provides, we hypothesize that it is equally plausible. The most recent sources have shown that the subscapularis tendon is not a single mass, but can consist of one to eight distinct tendinous parts (Zielinska et al. 2021c). In the event of rupture, therefore, it is possible that only one tendinous slip is torn, not the entire tendinous insertion. In such cases, non-operative treatment is usual. However, for more advanced ruptures, a reconstructive operation is required (Zielinska et al. 2021c). We think that an autograft using the ASM (especially if it has two tendinous parts) could help in such a procedure. One would have to remove the ASM and then create suitable material in the shape needed. In our opinion, this would help to maintain the efficiency and function of the SM; which is important, because only the SM is responsible for internal rotation in the rotator cuff.

This seems promising, but the procedure would have limitations. The first is that the ASM is a really rare structure, so it could be used in only a few cases. In consequence, this method will be not widely practiced. The next limitation is that the potential ASM should be detected before the operation, so we would need to use ultrasonography or another imaging method. However, there is no certainty that we would detect an additional muscle even if the patient has one. Therefore, we can conclude that this method would be unprofitable for many surgeons.

There is another possible advantage, however: the ASM in the present case originated on the lateral border of the scapula, but it was also connected to the fascia covering the SM. Connecting with the fascia implies that the ASM could transfer shrinkage and contribute to tension in the fascia, and therefore, also in the SM (Zielinska et al. 2021c). Moreover, the muscle in the present case was inserted via a tendinous slip to the capsule of the shoulder joint, helping to improve stabilization. The distal attachment of the ASM contributes to limiting the superior migration of the humeral head and maintaining its anteroposterior position. It is, therefore, involved in providing dynamic and passive stability to the glenohumeral joint (Kellam et al. 2019).

However, better stabilization can be associated with limitation of precise movements (Arias-Martorell 2019). To confirm this, biomechanical studies are necessary. This could be difficult because, as mentioned above, the ASM is a very rare structure; so even if this research is done, it will involve too small a sample for any conclusions to be drawn.

## Conclusions

The ASM is a very rare structure. It is usually connected with some kind of neuropathy, but not all types predispose to this. The muscle could possibly be used for an autograft when a SM rupture is treated. Moreover, it is associated with better glenohumeral joint stabilization, but at some cost to the precision of movements.

## Data Availability

Please contact authors for data requests (Łukasz Olewnik PhD—email address: lukasz.olewnik@umed.lodz.pl).
